# Connectivity for Healthcare and Well-Being Management: Examples from Six European Projects

**DOI:** 10.3390/ijerph6071947

**Published:** 2009-07-06

**Authors:** Maged N. Kamel Boulos, Ricardo Castellot Lou, Athanasios Anastasiou, Chris D. Nugent, Jan Alexandersson, Gottfried Zimmermann, Ulises Cortes, Roberto Casas

**Affiliations:** 1 Faculty of Health and Social Work, University of Plymouth, Drake Circus, Plymouth, PL4 8AA, Devon, UK; E-Mail: athanasios.anastasiou@plymouth.ac.uk; 2 Telefónica I+D, Parque Technológico Walqa, Edif. 1, Ctra Zaragoza 330 km, 58 Huesca, 22199 Spain; E-Mail: rcl@tid.es; 3 School of Computing and Mathematics and Computer Science Research Institute, Faculty of Computing and Engineering, University of Ulster at Jordanstown, Shore Road, Newtownabbey, Co. Antrim, BT37 0QB, Northern Ireland, UK; E-Mail: cd.nugent@ulster.ac.uk; 4 The German Research Center for Artificial Intelligence - DFKI GmbH, Stuhlsatzenhausweg 3, 66123 Saarbrücken, Germany; E-Mail: janal@dfki.de; 5 Access Technologies Group, Wilhelm-Blos-Str. 8, 72793 Pfullingen, Germany; E-Mail: zimmermann@accesstechnologiesgroup.com; 6 Artificial Intelligence Section (IA), LSI - Departament de Llenguatges i Sistemes Informàtics, Universitat Politècnica de Catalunya, Edificis C5-C6, Campus Nord, Jordi Girona 1-3, E-08034 Barcelona, Spain; E-Mail: ia@lsi.upc.es; 7 TecnoDiscap Group, University of Zaragoza, Maria de Luna 1/3, 50018 Zaragoza, Spain; E-Mail: rcasas@unizar.es

**Keywords:** telehealthcare, telehealth, telemonitoring, telecare, homecare, well-being and lifestyle management, wireless body area networks, assistive technologies, domotics, eHealth, Internet, older people

## Abstract

Technological advances and societal changes in recent years have contributed to a shift in traditional care models and in the relationship between patients and their doctors/carers, with (in general) an increase in the patient-carer physical distance and corresponding changes in the modes of access to relevant care information by all groups. The objective of this paper is to showcase the research efforts of six projects (that the authors are currently, or have recently been, involved in), CAALYX, eCAALYX, COGKNOW, EasyLine+, I2HOME, and SHARE-it, all funded by the European Commission towards a future where citizens can take an active role into managing their own healthcare. Most importantly, sensitive groups of citizens, such as the elderly, chronically ill and those suffering from various physical and cognitive disabilities, will be able to maintain vital and feature-rich connections with their families, friends and healthcare providers, who can then respond to, and prevent, the development of adverse health conditions in those they care for in a timely manner, wherever the carers and the people cared for happen to be.

## 1. Introduction

With the ageing population increasing across its member states, the European Union has undertaken a number of research initiatives around safeguarding technologies to assist the elderly. Targeted multidisciplinary European programmes have already been established to encourage integration between different basic disciplines and serve as strong drivers for the whole field of research and service development around ageing.

These projects operate in a challenging environment, producing innovative ideas to support the needs of the sensitive group of elderly people. They also operate in the perspective of a relatively young and highly complex telehealthcare market.

Older people are more likely (than younger age groups) to suffer multiple health conditions, chronic physical diseases and mobility limitation, often with concurrent mental and cognitive disorders, all of which requiring constant attention and care. The task of caring for these people is often shared by the family and/or other carers, a trend currently depicted by the increasing ‘elderly dependency ratio’ figures observed in many developed countries [1]. However, the elderly usually wish to remain independent and are likely to take the opportunity to migrate for prolonged periods of time to the milder climates of southern Europe [2], away from regular contact with their usual physicians. And even for those elderly people who choose to remain at home, the distance between them and their carers can become an issue. Routine visits by carers have been associated with lower mortality and a decrease in costly nursing admissions; however, the optimal number and duration of home visits is still an open problem [3]. Statistics and trends aside, all these people are unique ‘medical cases’, as well as being unique ‘whole persons’, with different individual needs that have to be covered, from the highly important aspects of disease prevention and lifestyle management to timely clinical care and follow ups.

The size of the population aged between 65 and 80 + years in Europe (EU-27) today is 80 million senior citizens [1], with a doubling of this figure forecasted by 2050. The life expectancy has already been rising on average by 2.5 years per decade and the number of ‘oldest old’ aged > 80 is expected to grow by 180% by 2050.

These trends are strong incentives for research institutions to produce high impact eHealth research, but there is also a corresponding, very large and flourishing market for relevant products and enterprises. Current technological advances have resulted in a set of technologies, namely the ‘Assistive Technologies’, proposing solutions to the issues of mobility, communication, remote monitoring and timely access to patient specific information. Application of these technologies in preventative healthcare is potentially beneficial for the elderly, but can also help in making provisions for ‘equal opportunities’ for people from all other age groups suffering from various physical and cognitive disabilities in all aspects of their daily living.

Due to these reasons, the field of eHealth is currently the subject of intense research and commercial development. Although there is no single “killer application” yet, a common pattern in the structure of existing systems can be discerned.

The objective of collecting and analysing multiple types of data from users, who can well be mobile and outdoors, is achieved first by establishing a ‘Wireless Body Area Network’. Currently an actively researched field, ‘Body Area Networks’ (BANs) are a natural extension of the ‘Personal Area Networks’ established by Bluetooth devices when applied to sensors collecting data from the human body. In fact, Bluetooth (http://www.bluetooth.com/) is the preferred short range wireless connectivity standard for this type of applications, though not the only protocol in use. A good commercial example of a BAN is Toumaz’s Sensium Life Pebble (http://www.ti.com/vitalsigns and http://www.toumaz.com/public/page.php?page=sensium_intro), a short-range-wireless-enabled, ultra-low power vital signs and Electrocardiogram (ECG) monitor offered in a miniature form factor that seems to be the most advanced example (miniaturisation-wise) of this class of sensors today.

Biomedical data collected off patients can be complemented by geographical (subject’s location) information, at least as captured by Global Positioning System (GPS) devices. These are becoming increasingly common, with major countries and federations planning to launch their own satellite systems, and electronic components for location sensors becoming so small that they can be easily integrated in a range of mobile devices and be carried around everywhere and anytime. The integration of GPS (and/or other positioning/location-aware technologies), with the ability to cross-reference the position of a user with the acquired vital signs data, has paved the way for a range of very useful applications today especially for the elderly, from simple localisation of patients with impaired memory function (e.g., wandering Alzheimer’s patients) to more complex applications for the objective estimation of activity and lifestyle, which is frequently a risk factor for patients with heart conditions and obesity [4].

This wealth of data produced by Wireless BANs needs to be collected in time and analysed to produce useful information relevant to the user’s condition. To this end, advances in semiconductor and wireless Internet technologies and in mobile operating systems have contributed to mobile phones, Personal Digital Assistants (PDAs) and other similar general purpose devices becoming more computationally powerful, feature rich, energy efficient, and most importantly cheaper, which makes them an ideal platform for eHealth applications such as Personal Health Management Systems (PHMS) [5]. Current examples of such systems are MobiHealth (http://www.mobihealth.org/) and HealthService24 (http://www.healthservice24.com/). Having powerful mobile devices that can handle both the local processing of sensor data and the secure transmission of specific information and events over the Internet only when it is absolutely necessary to do so is also significant for user privacy.

Establishing a BAN and connecting it to a powerful mobile device (data logger) is clearly essential, but applications can offer much greater impact for the benefit of user’s health if they are able to connect to the “outside world”, and more specifically to the Internet. Wireless communications have played and continue to play an important role in establishing these “outside world” links, enabling the delivery of feature rich content and feedback between users and between patients and their remote carers.

Advances in technologies such as GSM (Global System for Mobile communications), GPRS (General Packet Radio Service), 3G/WCDMA (third generation telecommunication hardware standards and technology for mobile networking/Wideband Code Division Multiple Access), HSPA/HSDPA/HSUPA (High Speed Packet Access/High-Speed Downlink Packet Access/High Speed Uplink Packet Access), Wi-Fi and WiMAX (Worldwide Interoperability for Microwave Access), and the associated communications protocols that have become an integral part of many mobile devices today, provide a layer of fast and reliable communications, free of the limitations of wires, upon which engineers can build further value-adding applications. Deployment of high speed wireless networks is already taking place across Europe, contributing to lower access costs and keeping users connected all the time. (Advances in these wireless technologies are also potentially beneficial for less advanced (developing) countries, as they can be deployed to quickly provide network access to remote areas.).

Systems that are generally based on this three-prong framework (BAN, mobile data logging/analysis device, and Internet connectivity) are usually complemented by stationary systems at the user’s residential home environment, widening the types of data that can be monitored, and also raising suitable alarms to prevent actions that could lead to accidents such as forgetting a heater on. These additional data range from weight measurements through a simple Bluetooth enabled scale to more complex biochemical parameters obtained through urine samples. Miniaturised sensors are also finding their way into household appliances enabling their easier management and making them safer. Short range wireless technologies have enabled the easy and quick deployment of such home systems, even in older houses.

This ecosystem of connected devices, systems and services has a strong potential in positively affecting the health of its users, as it constantly watches over them to prevent adverse health conditions or any event that could lead to such conditions, and also provides them with timely, relevant information about their health, including health education and lifestyle management material. This helps in getting the patients “in the loop” by making them more knowledgeable and aware of their health condition, and better equipped to safely assume responsibility for their own self-care.

Simple applications providing reminders to reinforce the impaired memory abilities of older patients can help them keep up with their medication. Such applications are significant, particularly for elderly patients at the early stages of dementia. Another typical example of PHMS applications is technology for Type II diabetics that can regularly monitor and assess, both in the short and long terms, their blood sugar levels and diabetes control status, instead of the patients just relying on the conventional (and much less frequent) face-to-face assessment visits to their clinicians (e.g., the technology developed by the M2DM—‘Multi Access Services for Telematic Management of Diabetes Mellitus’ European project: http://www.labmedinfo.org/research/m2dm/m2dm.htm and [6]).

Enabling feature rich communications between users can play a significantly positive role in the social and mental health well-being of the chronically ill, mobility restricted, lone and old, who can now keep in touch and socialise through suitable online social network applications with others who have similar problems, learn about their condition, share their concerns, and thus feel more safe and less stressed and isolated, thanks to peer support. Such applications can also prove useful in actively engaging healthcare providers with their target communities of users, ensuring (or educating and providing patients with suitable, user-friendly online resources to check) that any shared and exchanged health/medical information is evidence-based, valid and useful rather than hearsay.

Other advanced hardware solutions can reliably detect falls and automatically contact an emergency centre in a timely manner when a fall happens, which can help reduce fatal and costly complications of bone fractures. Besides the obvious financial benefits of such solutions due to the high total cost of healthcare for falls [7], older people relying on them can now feel more confident to live independently and to maintain regular levels of physical activity, both indoors and outdoors, which is very beneficial for their health and well-being (and in the prevention of osteoporosis and fractures).

Having these communications links and monitoring solutions in place will gradually transform the classic consultation and care paradigms, enabling healthcare providers and carers to check on their patients’ health by means of more frequent and cost effective “virtual” visits. The next section of this paper will explore the innovative research currently undertaken by six European projects towards realizing the benefits of this vision of a connected healthcare system.

## 2. Examples from Six European Projects

### 2.1. CAALYX: Complete Ambient Assisted Living Experiment

**Figure 1.**
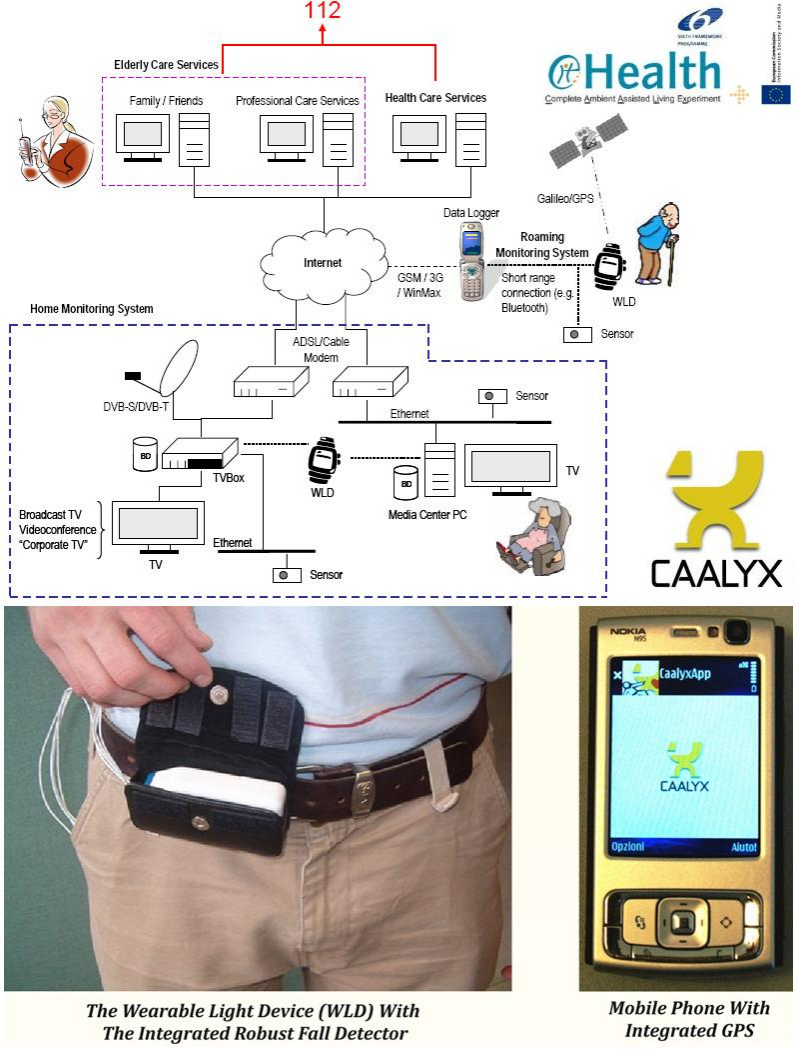
CAALYX’s system diagram. In the final system prototype presented in March 2009, the WLD (Wearable Light Device) takes the form of a waist-belt mounted device that communicates with a mobile phone (data logger) running the CAALYX application.

CAALYX (01/2007-12/2008 - http://caalyx.eu/) is a two-year eHealth project funded by the European Commission under FP6 (Sixth Framework Programme) [8]. It integrates the efforts of eight research, telecomm/industrial and end-user partners from six European countries, with the common goal of monitoring the health status of elderly users 24 hours a day, seven days a week, for the purpose of predicting/detecting any unfolding adverse health conditions and preventing complications before they develop, all while respecting users’ privacy and personal life needs. In the unfortunate event that an adverse condition arises, the system relays a high priority message to an emergency service (e.g., 112), including the geographic position and clinical condition of the elder user. In order to achieve these objectives as unobtrusively as possible, the service is composed of three distinct subsystems (Figure 1):

- The Mobile (Roaming) Monitoring Subsystem:
  - Collects and monitors key vital signs and detects adverse health events and falls when users are outdoors;
  - Facilitates efficient communications between the user and his/her caretakers and family in the event of an emergency when the elder person is away from the home environment.
- The Home Monitoring Subsystem:
  - Monitors users while at home and also helps keep them in touch with their family and caretakers;
  - Delivers classic services (television, videophone) and features rich Internet communications.
- The Caretaker’s Monitoring Subsystem (Elderly Care Services):
  - Provides concurrent monitoring of a number of elders by specialised personnel in an efficient manner. The caretaker decides whether to promote a raised event to the emergency service (112) or not.

While at home, a series of smart sensors (some of them, e.g., body temperature and ECG sensors, are worn on the body as a BAN, and others, e.g., weighing scale, are not) connect wirelessly via Bluetooth to a media-centre Personal Computer (PC), which records and analyses user parameters such as body weight, body temperature, blood pressure, cardiac function, etc. An ‘always-on’ connection to the Internet keeps the user within reach of their caretakers and family.

When the user is outdoors (for example, shopping in the city), he/she remains watched over and connected by carrying, in addition to his/her GPS-enabled mobile phone, a Wearable Light Device (WLD) that continues recording and analysing key vital parameters (body temperature, electrocardiogram, respiratory rate, etc.), and is also equipped with a reliable Fall Sensor (a tri-axial accelerometer) and a Panic Button. In essence, the WLD complements the functionality provided by the user’s mobile phone, acting as an active Bluetooth-enabled ‘sensor peripheral’ for it, and providing specialised hardware that is normally not available in a common mobile phone.

In order to respect the privacy of elder users, all the data processing required to evaluate their health condition is taking place locally, either on the Home or the Roaming Monitoring Subsystems, and only the outcome of this intelligent processing is shared with external entities, such as the carers, when this is necessary, e.g., during an emergency or if a fall is detected.

This approach, of sending selected data only when needed rather than streaming all data continuously, not only preserves the elder’s privacy, but also helps prolonging the Mobile Subsystem’s battery life and making best use of its Internet bandwidth.

Caretakers and other trusted entities with suitable access rights can request data and other information through the Caretaker’s Monitoring Subsystem. Because of the nature of the monitoring service, it should be possible in a future iteration of the system for the Caretaker’s Monitoring Subsystem to have access to a patient’s Health Record data over a standardised connection with the national health and social care infrastructure/databases where these are available.

The monitoring solution proposed by CAALYX is amongst a currently small number of solutions that are able to monitor the well-being of individuals *both* at home and while they are mobile. Wireless communications have played an important role in making the system usable and most importantly portable. Bluetooth is used extensively for the communications between the sensors that compose a Wireless BAN, which means that the system looks more like a set of wearable items rather than a bulky and heavy wire harness that would put-off potential users. The connection maintained through the user’s mobile phone and the GSM network, along with the high speed Internet connection of the Home Monitoring Subsystem, ensure that the patient is always within reach in case of an emergency. In this case wireless technologies have enabled a shift in the patient-doctor relationship, minimising the need for routine visits without losing touch, and also contributing to the autonomy and confidence of the users, which is an important psychological factor for the elderly.

### 2.2. eCAALYX: Enhanced Complete Ambient Assisted Living Experiment

eCAALYX (06/2009-05/2012 – http://ecaalyx.org/) is a three-year project funded by the European Commission under the Ambient Assisted Living (AAL) Joint Programme (http://www.aal-europe.eu/). The project builds on the strengths of the infrastructure and functionality already developed in the CAALYX project (see above), and in fact, six of the participants in new eCAALYX project (Telefónica Investigación y Desarrollo, Spain; INESC Porto – Instituto de Engenharia de Sistemas e Computadores do Porto, Portugal; University of Plymouth [Enterprise Ltd], UK; University of Limerick, Ireland; Corscience GmbH & Co KG, Germany; and Fundació Hospital Comarcal Sant Antoni Abat, Spain) were members of the previous CAALYX project consortium, with four new participants joining them in eCAALYX (Fraunhofer Portugal; CETEMMSA Technological Centre, Spain [project coordinator]; Ev. Krankenhaus Witten GmbH, Germany; and TeleMedic Systems, UK).

eCAALYX’s objectives can be summarized as follows:

- Health monitoring of older and elderly persons with multiple chronic conditions, at home and on the move (the original CAALYX did not cover the health monitoring and management of older people with *comorbidity*).
- Improve the quality of life of elderly persons by increasing their freedom and safety.
- Prevent deterioration of the patient condition by providing continuous support, guidance, and relevant health education.
- Achieve all of the above goals by providing a solution that is *commercially viable*, acceptable by all users/stakeholders, reliable, long-term, flexible, scalable, and virtually maintenance-free in non-technical environments, thus suitable for real-world deployment.

Chronic cardio-respiratory diseases are a major cause of death and admission to hospital among elderly people in Europe and are often concurrent with a number of other chronic health conditions. During the course of such diseases, repeated decompensation episodes typically occur, leading to frequent and costly hospitalisation of patients.

eCAALYX provides a complete solution to improve the quality of life of elderly patients in such a situation, by monitoring their health condition, assessing their health risk, promptly detecting and controlling any decompensation episodes, and offering them focused education on leading a healthy lifestyle, so that their independent life at home can be extended and their hospitalisation or admission in nursing homes avoided for longer periods. This ‘(self-)management and prevention’ approach is based on a ‘health agenda’ concept that is available at home and also when the aged person visits alternative homes during holidays or when visiting relatives, even in other European countries.

**Figure 2.**
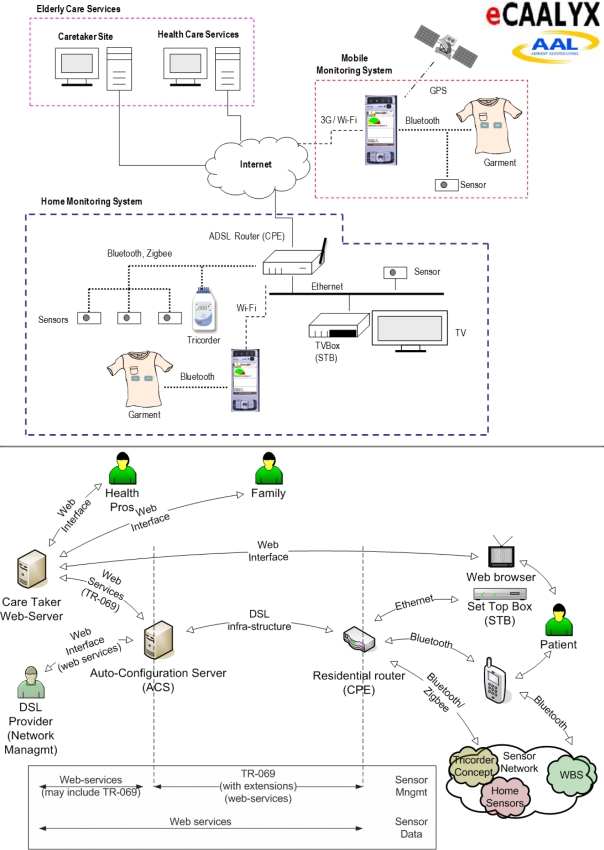
eCAALYX’s system diagram.

eCAALYX is composed of three main subsystems (Figure 2):

- The Home Subsystem, which includes Customer Premises Equipment (CPE), Set-top-box (STB)/interactive TV (to deliver health education and other functions), Tricorder and home sensors (those sensors that are stationary and not continuously worn on the body), all of them located at home;
- The Mobile Subsystem, which includes a “smart” garment, with all sensors integrated into a wireless BAN—Wearable Body Sensors (WBS), and a mobile phone;
- The Caretaker Site, which includes the Caretaker Server and the Auto-configuration Server.

The key features and main components of the three subsystems are described below.

eCAALYX will apply and extend standard network management standards, e.g., DSL Forum TR-069 (http://www.broadband-forum.org/), used by telecommunication service providers, to eHealth systems. Such interfaces and communications mechanisms can be used to remotely manage eHealth equipment, helping reduce operating costs, improve reliability and enhance user satisfaction. Integration of eHealth functionalities in standard (and low-cost) home networking equipment, namely DSL (Digital Subscriber Line) residential routers and set-top-boxes will reduce the equipment investment required.

The Tricorder device will combine the most relevant sensors for monitoring the prevalent chronic conditions and health risks in elderly people, such as hypertension, stroke, respiratory diseases and congestive heart failure. Thus sensors for the measurement of heart rate, ECG and pulse oximetry/SPO2 (Saturation of Peripheral Oxygen) will be included. By applying suitable processing and analysis algorithms to captured vital parameters, possible risk factors such as atrial fibrillation (an important risk factor for stroke), rise of blood pressure, progression of chronic obstructive pulmonary disease (COPD) can be detected. Inspiration effort/respiratory rate can be determined out of ECG measurements, and specifically out of the ‘pulse transit time’.

The basic sensor system of the Tricorder will be scalable; thus, interfaces for integration of additional sensors will be provided. Moreover, a Bluetooth interface will facilitate the linking of further Bluetooth-enabled medical devices such as weighing scales, blood pressure meters or glucose meters.

Algorithms and improved sensors/accelerometers will also be developed and evaluated for Activities of Daily Living (ADL) event detection (for falling, walking, climbing stairs, sitting, etc.), with improved sensitivity, specificity and power consumption, and these events (with alert status) will be forwarded optimally to the Home and Caretaker Subsystems.

This ADL/patient mobility component of eCAALYX will include algorithm development for gait variability measurement and for transmission of gait variability measures for data mining of fall risk. Based on the identified importance of energy expenditure for mobility qualified physiological signs, more accurate energy expenditure measurements derived from ECG, skin temperature, GSR (Galvanic Skin Resistance) and accelerometer signals will also be explored.

The integration of GPS (including the European Galileo Satellite Positioning System, when it becomes available) will allow detailed patterns of mobility to be determined, which can be used to further qualify measurements, and will be used to determine, in conjunction with data mining techniques, if any deterioration in mobility is occurring.

Using conductive textile materials, a comfortable garment will be developed for improved usability, user acceptance and performance. It will have conductive areas which will be used as heart rate/ECG electrodes when in contact with the skin. Conductive fabrics allow electrodes to be smooth to the touch and comfortable. Moreover, using conductive fabrics for data transmission between the non-wireless parts of a BAN is a major improvement in a wearable system, replacing bulky electric wires by conductive textile ribbons. Solutions will be selected according to comfort requirements such as garment flexibility, elasticity and washability.

A standard GPS enabled smart phone (3G/UMTS—Universal Mobile Telecommunications System) running a completely autonomous software application, requiring no user interaction, will manage the personalised BAN, which comprises the garment and other user specific sensors, and continuously analyse sensor data in order to identify problem situations and alert health professionals. This software application will be a refinement of the one developed for the CAALYX project, the development this time being focused on areas such as reliability, network management (3G/Wi-Fi switching), improving remote and automatic configuration and management, and reducing mobile phone battery consumption.

A distributed, adaptable and scalable monitoring infrastructure will be implemented to allow for the continuous acquisition of the users’ sensors data and to provide these data in a meaningful and controlled way to the client applications on the Caretaker Server, the Home Subsystem and the Mobile Subsystem. This infrastructure will allow for the health professionals to easily configure software/virtual sensors for each user (‘observation patterns’ or schedules), using the set of available real and pre-existing virtual sensors, i.e., the user’s BAN, other sensors at home, the Mobile Subsystem (geo-positioning), relevant user data/history stored on the Caretaker Server and, eventually, data from the person’s national/central health and medical records.

Coordinating teams of healthcare professionals can be a major issue when dealing with patients with comorbidity, whose conditions require the expertise of, and input from, multiple specialists. The Caretaker Server will address this issue by providing appropriate means for coordination, e.g., an electronic health record and personalised forums for each patient, as well as direct and easy modes of communication between health professionals such as video-conferencing.

Besides enabling reactive actions in response to acute event alerts, the Caretaker Site will proactively use data mining techniques to correlate data from sensors, assess risk levels and help switching any corresponding ‘preventive actions’ in the ‘health agenda’ in a timely manner. Pattern detection via data mining of readings from multiple sensors (i.e., looking for normal and abnormal patterns, plus changes and trends over time) and the provision of effective reporting mechanisms to the caretaker will be implemented.

Contextualisation of body sensor data using ambient sensors in the home (RFID—Radio-Frequency Identification and PIR—Passive Infrared Sensors) will enhance the home mobility data of monitored patients by facilitating the linkage of these data to the person’s daily activities in the home. Data from GPS and weather stations will enhance the mobility data of the monitored patients outdoors. Factors such as when, where and to what extent the person is sitting, lying or walking will be examined, since these factors could be significant in assessing the health status of the patient.

Communications security and elder’s privacy are ensured by using standards recommended by HL7 (Health Level 7 - http://www.hl7.org/) and including ‘audit trailing’ capabilities to log ‘who has done what and when’. Standards developed under the Service Oriented Architecture (SOA) architecture will be applied. It should also be possible to make all relevant components of eCAALYX compliant with Continua Health Alliance (http://www.continuaalliance.org/) recommendations, which are based on ISO/IEEE 11073 and Bluetooth standards.

### 2.3. CogKnow: Helping People with Mild Dementia Navigate their Day

CogKnow (09/2006-08/2009 - http://www.cogknow.eu/) is a three-year project funded by the European Commission under FP6. It has 11 multidisciplinary participants (from seven European countries) with backgrounds in computer science, biomedical engineering and the clinical domain. The objective of the project is to develop a user-validated, cognitive prosthetic device and associated services for elderly people with mild dementia. Such a solution will have a tremendous impact on the quality of life and autonomy of persons with mild dementia and will potentially increase the period of time they can remain independent and living at their own home.

The project is focused on understanding the needs of patients with mild dementia of the Alzheimer type and on identifying how portable and configurable technological solutions can best help these persons to navigate through their day. Specifically, cognitive reinforcement prototypes are developed in order to help patients:

- Remember;
- Maintain social contact;
- Perform daily life and recreational activities;
- Enhance their feelings of safety.

**Figure 3.**
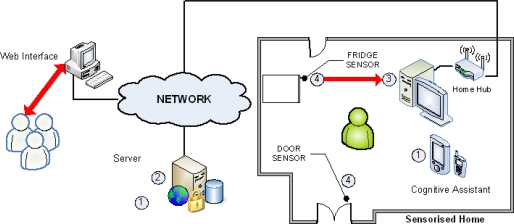
CogKnow’s system diagram.

The project efforts are driven by a strong user-centred focus [9]. Besides knowledge on needs in dementia identified by reviewing the literature, results from workshops and individual interviews with dementia sufferers and with their carers in three countries (The Netherlands, Sweden, and Northern Ireland) were used to formulate the functionality of the technical solution. Based on a ranked analysis of the needs and preferred ICT (Information and Communication Technologies)-solutions following workshops within the project, it was possible to identify top-priority services and the functional requirements necessary to specify the main technical components of the CogKnow system. These are depicted in Figure 3 and include:

- The Home-based Hub:
  - Responsible for the collection of all information pertaining to the activities of the person suffering from dementia within his/her home (through suitable sensors in the home environment), and for relaying this information to the CogKnow Web server. It is a stationary device located at a fixed position within the person’s home and featuring a 17 inch LCD (Liquid Crystal Display) touch screen.
  - User interaction is facilitated through a graphical user interface which can be personalised to meet user’s preferences and deliver the necessary reminder messages. It also offers a picture dialling service, supports activities such as listening to the radio or to pre-stored music, and contributes to enhanced feeling of safety through the provision of warnings when doors are left open or devices are left on.
- The Mobile Cognitive Prosthetic:
  - Its purpose is to mirror the services offered by the Home-based Hub, so that they may also be accessible from anywhere within (or outside) the home environment and not just through the large LCD screen. The Mobile Cognitive Prosthetic (or Assistant) is a mobile device employing standard smart phone technology with a 2.8 inch touch screen and a Windows Mobile operating system.
  - Within the home environment the mobile device communicates with the stationary Home-based Hub device via a Wi-Fi network. Once outside home, the mobile device utilises GPS technology to support users if they become lost and require support to ‘take them back home’.
- The Web-based Server
  - Provides care management for the person with mild dementia. This is the main information repository of the entire system, where users’ information, their needs and recorded details of their activities of daily living are stored. It also provides the means for the carer to configure and schedule patient reminders.
- The Sensorised Home Environment
  - This includes both sensors and actuators. Sensors are attached to domestic appliances (e.g., the fridge) and doors, and communicate wirelessly with the Home-based Hub. All components within the system are integrated via a two part communication model. The first part relies on an underlying TCP/IP (Internet Protocol Suite: Transmission Control Protocol and the Internet Protocol) network used to transfer XML (Extensible Markup Language) and Ivybus protocols (http://www.tls.cena.fr/products/ivy/about.html) [9]. The XML facilitates duplex communication between the Home-based Hub, the mobile device and the server. The Ivybus supports the Home-based Hub access to sensor control and data via calls to the sensor network. The second component relies on the power infrastructure cables in the home environment as its primary communications channel using the X10 protocol (a communications protocol for remote control of electrical devices over standard household wiring—see: http://tinyurl.com/oykv93). This is the main underlying component used to support the actuators within the system.

System evaluation focused on user friendliness, usefulness and efficacy of the full CogKnow solution from user, technology and business perspectives [9,10].

By harnessing technology and developing suitable “cognitive prostheses” to help people with dementia remember, maintain social contact, and carry on with their daily life activities, Cogknow has the potential to improve the overall quality of life of these people, enhance their feelings of safety and social integration, and assist them in living independently at their own homes for longer periods of time. (The “cognitive prostheses” developed in CogKnow include audio-visual reminders and guidance messages on both a TV screen located in the living room and a handheld mobile phone to prompt the person to call his/her family, prepare his/her meals or secure the house’s front door. The system also checks, through sensors embedded in the home environment, that these actions have been properly completed by the user. An online demonstration video explaining CogKnow’s vision in a practical home environment setting is publicly available at http://www.cogknow.eu/documents/dissemination-material/project-video.)

### 2.4. EasyLine+: Low Cost Advanced White Goods for a Longer Independent Life of Elderly People

**Figure 4.**
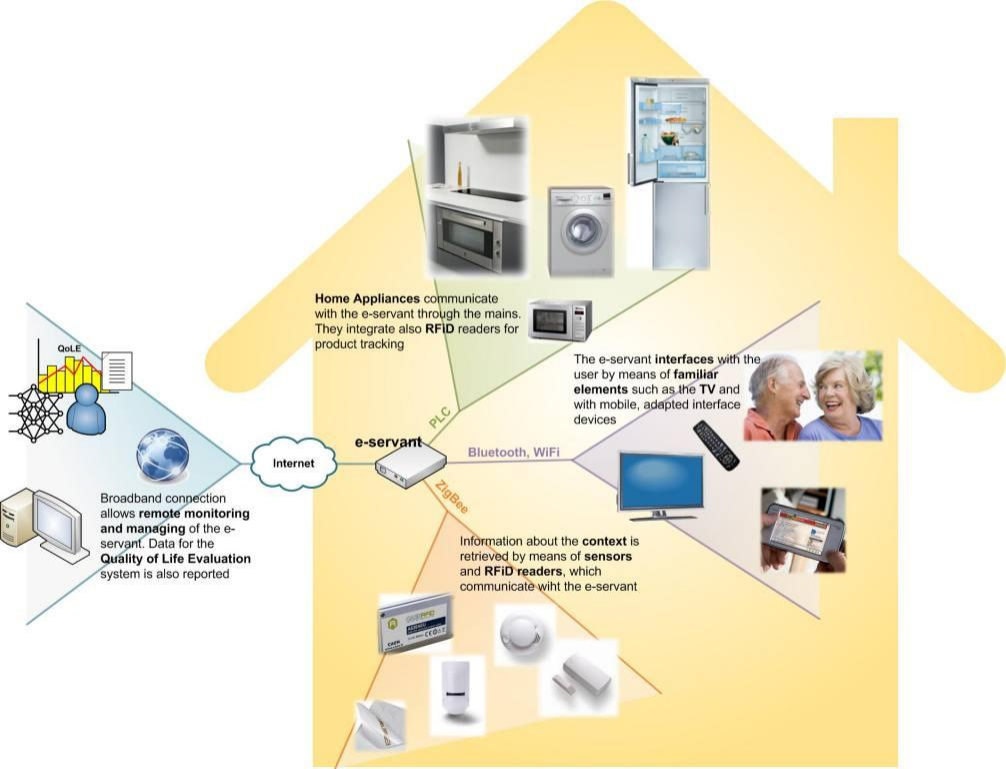
EasyLine+’s system diagram.

EasyLine+ (01/2007-06/2009 - http://www.easylineplus.com/) is a two-and-half-year project funded by the European Commission under the FP6 eInclusion programme. Six partners from three European countries have developed prototypes suitable for the market of advanced white goods to support elderly people with or without disabilities in carrying out a longer independent life at home, and to compensate for any loss of physical and/or cognitive abilities they might have in the kitchen. This is achieved by bringing ambient intelligence to the kitchen environment. The project’s architecture is shown in Figure 4.

Within the modern kitchen environment, white goods are perhaps the most important elements, whether they are “intelligent” or not. To achieve EasyLine+’s goals, new ambient “intelligence” and interfaces have to be introduced across the whole system, but this does not necessarily imply that existing white goods have to be made “smarter” or to incorporate new adapted interfaces. Such radical modifications would only increase their unit price, make their installation harder (as adapting their functioning to a user’s particular case would require custom configuration), and consequently hinder their market penetration.

Instead of relying on new “smart” appliances with accessible interfaces, EasyLine+ features a ‘central intelligence system’ that is aware of the status of all white goods in the kitchen, able to control them, and also able to interact with the user. This essentially means giving home appliances the capacity to communicate.

Besides the developments related to white goods, EasyLine+ introduces diverse sensors that are usually used for context awareness tasks. Some sensors are already used inside today’s kitchens (temperature, fire, smoke, and flooding), while others are not yet common (presence, door opening, etc.). The project team have also considered using RFID readers to identify clothes and food. The use of RFID and EPC (Electronic Product Code - http://www.epcglobalinc.org/) can provide useful information about what is in the washing machine or fridge, what does the user want to eat or have just bought, etc. Food information can be used to inform the elderly person about any food ingredient that is missing, which food is nearing or has reached its expiry date, which meal the user may prepare and eat, etc. Cloth information can be helpful in determining which washing programme is best suited for clothes introduced in the washing machine.

A Human-Machine Interface (HMI) is managing user interaction and is of key importance to the system. HMI devices should be easy to use and suit any kind of user, with the ability to change the interface according to various user profiles. They do not necessarily require powerful processing capabilities or a high data storage capacity, but should have a standard communications interface. There are many different types of mobile clients that can be used to interact with the system such as PDAs, smart phones, ultra-mobile PCs and others. This means that the user can carry them around the house and be able to monitor the house appliances wherever he or she might be. Fixed devices such as desktop computers, digital TVs or devices in the form of embedded ‘control panels’ in the actual appliances can also be used as control interfaces.

The system as a whole is called e-servant and is aware of its environment and users. The “intelligence” of white goods is enhanced by offering new functions that facilitate their use. The system adapts to any disabilities or preferences the user might have by analysing all the data it gathers and extracting relevant information that can be used to adapt the system to suit the needs of its user. In the case of an emergency, the system can send a warning message to a remote care centre.

E-servant is also the ‘coordinator’ with which all the other system components communicate. In order for the system’s operation to be transparent to the user, the different building blocks communicate with each other using various modes of communication:

- Power Line Communication (PLC) over the mains wires is used to get the status of appliances and to control them. PLC is the best option for white goods because they all have to be mains powered and there are already European standards being promoted in this respect. Moreover, no configuration is needed to install a new appliance.
- A ‘wireless sensor network’ supports all the sensors in the kitchen and also the acquisition RFID data from corresponding readers. This sensor network is operating over ZigBee (http://www.zigbee.org/), as this is becoming the de-facto standard for home environments.
- Bluetooth and Wi-Fi are employed for the communications between the interfaces due to the widespread availability of these technologies in personal computers and mobile devices.
- Finally, communication with the “outside world”/the Internet (e.g., to send to social services or family information about the user’s quality of life, as well as alarms about possible technical problems or any appliance maintenance needed, etc.) is carried out using standard protocols such as DSL (Digital Subscriber Line) or Wi-Fi.

### 2.5. I2HOME: Intuitive Interaction for Everyone with Home Appliances based on Industry Standards

I2HOME (9/2006-8/2009 - http://www.i2home.org/) is a three-year project funded by the European Commission under the FP6 eInclusion programme. The project consortium is composed of nine partners from five European countries, with competences ranging from technology development to accessibility expertise and experience with persons with special needs. The project builds on two main pillars:

- The implementation of the ISO/IEC 24752 ‘Universal Remote Console’ (URC) standard;
- The development of user interfaces for disabled persons following a user-centred design methodology.

Current research and technology provide the necessary building blocks for realising “smart” home environments, heralding an era of new interaction paradigms for persons with special needs. However, most of this technology is either proprietary or incompatible with some existing components. Moreover, current user interfaces are targeting advanced users by offering them to go in detail through every feature of the service or appliance. This can quickly become overwhelming, not only for persons with cognitive disorders but even for ordinary users wanting to complete a quick task. At the same time, knowledge on how to design accessible user interfaces is expanding but the corresponding technologies are not adequately marketed and implemented in practice. There is clearly a need for an open universal platform for connecting interaction devices with appliances and for services that provide more flexible user interfaces.

**Figure 5.**
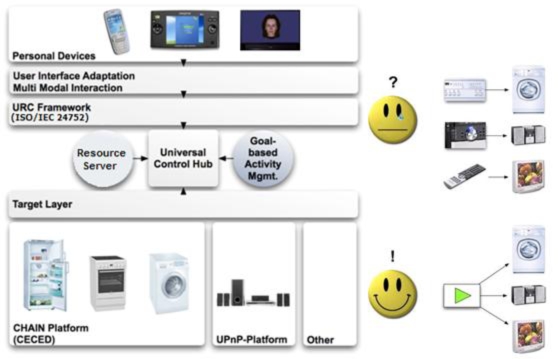
The Universal Remote Control standard. CECED = *Conseil Européen de la Construction d'appareils Domestiques*, which represents the household appliance industry in Europe. UPnP = Universal Plug and Play--see http://www.upnp.org/.

These requirements are met by the ISO/IEC 24752 URC standard depicted in Figure 5. The architecture includes a hub for connecting various appliances and services with exchangeable, pluggable user interfaces, as well as Resource Servers that enable the sharing of various resources, such as target adaptors or remote user interfaces. The ability to switch user interfaces and the presence of Resource Servers are highly innovative concepts and present a major advantage over other approaches.

I2HOME followed a user-centred design methodology to develop four distinct user interface prototypes for the cognitively disabled, Alzheimer’s patients, the elderly, and sensory impaired patients. The main functionality of these user interfaces enables the interaction with main stream technology such as Siemens Serve@Home platform (http://www.se-presse.com/download.php?pfad=downloads/en/19-1012-0501e.doc) and Google Calendar (http://calendar.google.com/). Further work will focus on streaming technologies for the purpose of video conferencing. This is an important and necessary prerequisite to support a level of social networking functionality.

The project is an important step towards the creation of a European ecosystem for URC technology and is already in close collaboration with other projects of the European Commission eInclusion programme, acting as a technology supplier to them.

I2HOME has the potential to make the following contributions to eHealthcare through the innovative use of URC technology:

- Overcome the ‘one-size-fits-all’ limitations through pluggable user interfaces;
- Adapt and customise the services and associated user interfaces via shared resources;
- Develop next-generation user interfaces with simplicity and ease of use in mind through task-orientation and natural language interaction;
- Offer to other projects and manufacturers the ability to re-use the Universal Control Hub product for the development of a standards-based middleware architecture for the digital home of the future.

The worldwide coordination of the URC technology, which stands at the heart of the I2HOME project, is performed by the URC consortium (http://myurc.org/) and the Trace Center (http://trace.wisc.edu/). Details of the first official URC Resource Server can be found online at http://myurc.org/tools/ResourceServer.php/index.php.

I2HOME’s ultimate goal is to provide a unified and accessible user interface to common home appliances and consumer electronics based on industry standards. This approach should make it much easier for people with cognitive disabilities and older persons to operate these devices, e.g., by using an intuitive interface running on an Apple iPhone, without having to learn and remember a different and complex operating interface for each appliance separately, thus helping these persons live independently at their own homes for longer periods of time. An online video demonstration of some aspects of the technology adopted in I2HOME is publicly available at http://www.youtube.com/watch?v=wElYM_nb7gI.

### 2.6. SHARE-it: Supported Human Autonomy for Recovery and Enhancement of Cognitive and Motor Abilities Using Information Technologies

The aim of SHARE-it (01/2007-12/2009 - http://www.ist-shareit.eu/shareit) is to contribute to the development of next generation intelligent and semi-autonomous assistive devices for persons with cognitive and motor disabilities [11]. These persons can then reach a sufficient degree of autonomy allowing them to reside in their homes as long as possible, with a high level of safety and comfort. This should also increase their quality of life, and, at the same time, delay their move to specialised care institutions.

In particular, the objectives of SHARE-it are:

- To explore the benefits of the concept of situated intelligence by building elements that can enhance the autonomy of the target users in their daily lives, allowing them to stay in their preferred environments;
- To investigate and implement innovative forms of shared autonomy;
- To develop appropriate add-ons to standardised technologies to provide ubiquitous sensing, computation and assistance;
- To build multimodal and adaptive interfaces for the target user group;
- To assist existing caretaking services as effectively as possible.

Novel technologies promise radical advances in the support of Europe’s elderly citizens with disabilities. Assistive engineering and design is a field at the intersection of technology, natural sciences, humanities, social sciences and medicine. Assistive Technologies are increasingly gaining importance in a society whose demographics are rapidly changing. The average life expectancy in the European Union is one of the highest in the world, and is continuing to rise [1]. The percentage of persons suffering from disabilities is rising simultaneously, with data showing that half of the elderly population is living with some form of impairment. Clearly, human resources will not be sufficient to assist this growing population group in their daily life needs, and solutions of the kind proposed by SHARE-it are expected to play a key role in this respect.

**Figure 6.**
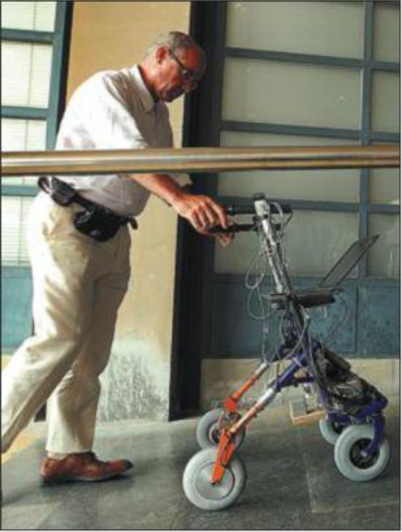
The iWalker prototype (see: http://www.youtube.com/watch?v=sCTOvM0pl48).

SHARE-it is achieving its goals through the use and/or development of:

- The semi-autonomous wheelchair Rolland III which functions safely indoors and outdoors in appropriately set-up environments;
- The semi-automated guided platform Spherik: can serve as a mobile docking station for a patient’s wheelchair and uses innovative spherical casters to enhance flexibility and mobility [12];
- The Intelligent Walker Platform or iWalker [13] (Figure 6), which reuses the existing assistive technology developed for Rolland and Spherik.

All three mobile platforms are able to:

- Detect a patient’s position in their homes through a specific monitoring system;
- Adapt autonomously to the user’s assistance requirements through a tailored cognitive model based on the interpretation of bio-sensor data and on disability profiles assessed by a medical team;
- Monitor and interpret clinical information (such as different bio-patterns and stress levels) and send it to the caregivers.

The “intelligent” infrastructure of SHARE-it sets a common framework for underpinning reasoning, decision-making, learning, communication and other services, and for embodying these in a set of generic software components that are of high quality, scalable and robust. The component set has been designed for incorporation into assistive systems. Assistive technology is focused on monitoring, information provision and alarm distribution, as well as mobility assistance, and by serving this field, SHARE-it and similar solutions are expected to have not just scientific and technological values, but also social and commercial impacts in the near future.

Robotics, artificial intelligence and information and communication technologies such as those included in the intelligent walker (i-Walker) developed by SHARE-it can compensate for the loss of sensory, motor and cognitive functions caused by natural ageing processes and by disease in the elderly. SHARE-it’s i-Walker goes a step beyond conventional walkers by being able to communicate with the user, think for itself and react to the environment. The device can be activated by means of simple voice commands given by the user, e.g., “take me to the kitchen”, and is thus suitable for use in medical rehabilitation scenarios (online videos of i-walker and other project demonstrators are available at http://www.ist-shareit.eu/shareit/material).

## 3. Discussion

In this paper, we described six research projects, showcasing on-going European efforts towards realising the full benefits offered by telehealthcare, assistive technologies and domotics, and underlining the importance of wireless communications in delivering highly usable systems. There are many other European-funded projects with similar and overlapping objectives, e.g., DREAMING (Elderly-friendly Alarm Handling and Monitoring - http://www.dreaming-project.org/) and PERSONA (Perceptive Spaces Promoting Independent Aging - http://www.aal-persona.org/), but we chose to present only projects we are personally involved in as researchers.

There are still many challenges and barriers on the way to the full market penetration and commercial adoption of the solutions researched and developed in these projects. These ‘challenges from prototype to product’ and barriers are well summarised in a recent SlideShare presentation by Kamel Boulos (http://www.slideshare.net/sl.medic/telehealthcare-promises-and-challenges) and in [14,15].

In the healthcare and social care domains, the benefits of ‘staying connected’ and ‘communicating’ cannot be underplayed, both for healthcare/social care providers and their consumers, and it is very likely that in the future, this vital connection and the systems that support it will become rather ubiquitous and as available as the telephone, for example, is today. The progress towards the realisation of this vision largely depends on the following three equally important factors:

- International/European policies;
- Stakeholder attitudes; and
- Technological advances.

Experience has shown that, due to the complex financial issues and interdependencies involved in national healthcare systems, obtaining political backing and support is crucial if one is to be able to reap and sustain the benefits of eHealth solutions at national level. Such high-level endorsement should cover the choice and enforcement of the right standards and protocols for interoperable systems and services at national level, ensuring that adequate policies are in place and taking any necessary regulatory or executive actions to enable the successful adoption of ‘connected eHealth’ at the various levels of the health and social care system. This includes, among other things, extending high-speed network infrastructure coverage to reach remote places and rural areas where eHealth can have the highest impact, but perhaps most importantly, communicating with, informing and educating all involved stakeholders about the benefits of eHealth to secure their long term support, involvement in the formulation, and acceptance of any proposed solutions.

This latter point is closely related to the ‘human factor’ in eHealth service provision, and to the attitudes of stakeholders, whether these are healthcare providers, patients, families or other carers. For quite a long time now, remote monitoring and networked access to personal information have been associated with negative perceptions of a ‘big brother’ situation. These negative perceptions have been reinforced by the security problems at all levels that marred many online applications in recent years. Fortunately, some trust in online communications is starting to be regained, albeit slowly, as evidenced by the growing number of novel personal eHealth services and location-aware applications on offer today such as Google Health (https://www.google.com/health), Google Latitude (http://www.google.com/latitude), Microsoft HealthVault (http://www.healthvault.com/), and Microsoft Vine (http://www.vine.net/), and also by the fact that the last five years or so have seen many European and US healthcare providers modernising their core ICT infrastructure and creating more robust platforms to support the next generation of wireless eHealth applications [16].

Current studies suggest that people over the age of 65 use mobile phones for very limited purposes, such as for calling or texting in emergency situations. They avoid using more complex functions. The major causes cited are displays that are too small and hence difficult to read; tiny handset buttons; too many, complex and sometimes unnecessary functions, with non-user-friendly menus and unclear instructions on how to find and use a certain function; and finally services that are too expensive to subscribe to. These causes are mostly usability related, and there are already some solutions designed to address them (see, for example, this mobile phone with simplified user interface and built-in emergency button that has been specifically produced with older and elderly people in mind http://www.emporia.at/shop/emporiaLife_ProductFlyer_GER.pdf). Mobile services are also increasingly becoming cheaper.

As technology broadens the wireless horizon for older people and those suffering from various physical and cognitive disabilities, it promises to enhance their quality of life and to provide them with new opportunities to live independently that were unthinkable of a few years ago. “Wireless technologies are growing in importance for users who are disabled and those who are not”, says Mike Jones, Shepherd Center’s vice president of research and technology (http://www.shepherd.org/research/staff.asp#jones - Shepherd Center is a private, not-for-profit hospital in Atlanta, GA, USA, devoted to the medical care and rehabilitation of people with spinal cord injury and disease, acquired brain injury, multiple sclerosis and other neuromuscular problems). Jones rightly believes that improving usability for people with disabilities will also improve usability for everyone (see: http://tinyurl.com/qzgo88).

Moreover, stakeholders’ attitudes and abilities are expected to gradually change when today’s generations of younger people who were born and educated in the modern information society (the Internet age and the era of personal and mobile computing) get older, as these people are more familiar and comfortable (than the current generation of senior citizens over the age of 65) with the concepts and modes of operation of ICT solutions, having used them most of their lives for study, work and leisure.

In the projects we have described, we have demonstrated how mobile phones can play an important role in helping older people and people with cognitive problems in many ways, especially with the increasing range of mobile phone-based applications and services on offer today, e.g., alerts and reminders, GPS and emergency calls. Mobile phones and associated services afford a sense of security to older people [17], and offer peace of mind to their families, who can feel assured that their elderly relatives are now reachable ‘anytime and anywhere’ and are being properly monitored regarding their health status and well-being. Such solutions can also reduce the burden on professional carers who do not have to continuously be in physical proximity to a person to watch over them.

Stakeholders constitute their own evolving market. During the last decade systems were commonly characterised by small scale, single application pilots and projects, while they are now increasingly dominated by larger, integrated multi-function applications and distributed services. The market for wireless based eHealth monitoring and diagnostics services is currently being driven by the incumbent healthcare provider desire to push key elements of the care process out towards the edge of their healthcare network [16]. The type of organisation that is building and hosting these eHealth services is also changing, with incumbent traditional healthcare providers ceding market share to ‘nextgen’ (next-generation) healthcare providers (or becoming themselves transformed to function as ‘nextgen’ providers). This evolution is in part driven by the growing use of both local and wide area wireless networking. Both ICT equipment vendors and mobile network operators regard healthcare as a rapidly expanding market for wireless technology and services.

Technological advances, especially those enabling faster, more reliable communications over various media and the incorporation of medical sensors and devices into clothing or jewellery (“smart” garments), are expected to radically transform the future of eHealth. The healthcare sector has for many years lagged behind the manufacturing and financial sectors in the adoption of automated processes [16], but this is now rapidly changing, thanks in part to the rapid advances in wireless and mobile technologies.

Two key factors seem to be driving today’s market for wireless eHealth platforms. The first factor is that by using these platforms healthcare providers can significantly reduce costs by implementing telemonitoring solutions for the early detection of health problems in patients, and subsequent reduction of complications and costly hospitalisations, while social care services can deploy assistive technologies and domotics to help people with cognitive problems live longer independently [18], which can again cut the costs of care on the long run. These types of services and technologies can also free up clinicians and other health and social care workers’ precious (and already constrained) time, by automating some of their routine tasks to help them better focus on their more demanding duties and serve more people more effectively. The second factor is the need for technology that can bridge Internet-based healthcare information portals, backend patient record systems and mobile-phone-based personalised eHealth applications [16].

This paper could only touch on some of the major features and *breadth* of the rapidly growing and massively interdisciplinary field of ‘telehealthcare, assistive technologies and domotics (in older people’s care)’, rather than cover the *depth* and fine details of this field, and the many overlapping issues that are associated with it, such as system and service design; industry standards and service interoperability; ethical considerations; and human interaction and socio-organizational factors [19]. The latter factors include users’ technology literacy and confidence in usage; technology functionality, usability, accessibility, and user acceptance [20]; contingency planning for system failures; and a range of organizational themes, including health economics issues and factors related to the different roles and specific concerns and needs of health and social care workers, as well as of “lay” carers.

Touching on just the breadth of this vast field and doing so in a comprehensible, balanced manner that can be appreciated by a wide range of readers from very different (and not necessarily technical) backgrounds was a big challenge due to the plethora of technologies that are involved in the subject. However, we hope that by “painting” and piecing together some of the ‘big picture’ (a bird’s-eye panoramic snapshot) of this important field and its current directions, we have managed to spark the readers’ imagination to start considering the potential synergies between the different technologies and systems we have sampled in this paper, and to highlight the need for, and importance of, developing comprehensive, *integrative* solutions and services in the future that can realise the benefits of all these technologies combined in the most efficient and effective ways to best serve the needs of older people and their carers.

## 4. Conclusions

Our brief six-project “sampler” demonstrates the importance of the recent advances and emerging developments in communications technologies, which are needed to act as the ‘binding glue’ in any future integrative solutions that link and transcend a wide range of health and social care services and communities of users.

A range of applications and services that could open up the healthcare market to a new generation of providers, such as home monitoring of the health status of the elderly and GPS-enabled phones to help Alzheimer’s patients when they have wandered around or become disorientated, have been prototyped, and some of these solutions are currently being marketed to patients and their carers. Enabling wireless technologies, such as Bluetooth, have been shown to be enormously useful in data transmission over short distances from fixed or mobile devices. Frail pensioners needing round the clock care could well become among the routine users of Bluetooth wireless technology in the near future. Some vital signs monitoring systems have already been developed that send sensor data via Bluetooth to a personal computer, which can be consulted (even remotely) by a doctor or care workers, enabling them to keep an eye on the health of the patient and spot any problems before they become life-threatening.
